# Draft Genome Sequence of the Biowarfare Simulant *Bacillus atrophaeus* Strain 930029

**DOI:** 10.1128/genomeA.00491-15

**Published:** 2015-06-04

**Authors:** Catherine Eng, Yann Blouin, Nicolas Ding, Guilhem Larigauderie, Vincent Ramisse, Céline Pujol

**Affiliations:** Division Biologie, DGA Maîtrise NRBC, Le Bouchet, Vert-le-Petit, France

## Abstract

We report here the draft genome sequence of *Bacillus atrophaeus* strain 930029. Strain 930029 shows evidence of drift, based on a comparison to the corresponding source strain publicly available today.

## GENOME ANNOUNCEMENT

The aerobic spore-forming *Bacillus atrophaeus* (formerly *Bacillus globigii* and *Bacillus subtilis niger*) (1, 2) has a long history as a model for Gram-positive organisms, and multiple biotechnological applications for this bacterium were recently reviewed (3). Among them, the species has played an important role worldwide in the biodefense domain as a simulant of anthrax (*Bacillus anthracis*), a high-priority biological warfare and bioterrorism threat. Multiple strains of *B. atrophaeus* have shown evidence of interlaboratory strain variation (4). The impact of such variations in standardized tests and evaluations of sampling and detection systems is questionable. The decades-long use of *B. atrophaeus* strain 930029 as a simulant for biodefense preparedness efforts in France motivated in-depth characterization by whole-genome sequencing (WGS).

WGS of strain 930029 was sequenced by using an Illumina MiSeq personal instrument and on paired-end reads with a read length of 300 nucleotides (nt). A total of 5,568,114 reads were generated, for a total of 1,236,558,746 bp. The reads were *de novo* assembled using Velvet version 1.2.07 into 131 contigs >1,000 bp, which suggests that this strain has a genome size of at least 4.05 Mbp, with a G+C content of 43.24%. The *N*_50_ contig length was 66,438 bp, with the longest being 192,113 bp. The average coverage for the contig sequence of strain 930029 was 101×. Using BioNumerics version 7.1, the sequence reads were mapped to the complete reference genome sequence of *B. atrophaeus* 1942 (accession no. CP002207), and a 16-kb deletion (positions 208228 to 224482) was identified in strain 930029. Multiple gene deletions affect hypothetical proteins and two genes encoding for RapK phosphatase and for an inner spore coat protein. Multiple (23 silent) single-nucleotide polymorphisms (SNPs) were also detected. Historically, strain 930029 was acquired from Collection de l’Institut Pasteur (CIP) (Paris, France) as CIP77:18 (batch “kia”) in 1993. The publicly available strain kindly provided in 2013 by CIP (batch “15409_3”) has been also sequenced; 6,528,908 reads for a total of 1,193,477,765 bp were generated. The reads were *de novo* assembled into 151 contigs >1,000 bp, which suggests that this strain has a genome size of at least 3.89 Mbp, with a G+C content of 43.74%. The *N*_50_ contig length was 64,872 bp, with the longest being 273,228 bp. The average coverage for the contig sequence was 69×. This strain is not missing the 16-kb fragment previously identified in strain 930029 and is discriminated from strain 930029 based on 19 SNPs. Therefore, strain 930029 presents features different from the two other isolates of the *B. atrophaeus* species, 1942 and CIP77:18, such as the conspicuous absence of genes involved in extracellular peptide signaling and endospore development. Despite this deletion, strain 930029 is easy to grow and sporulate *in vitro*. This raises the question of strain variations during long-term propagation and of standardization issues for tests and evaluations in biodefense.

### Nucleotide sequence accession number.

The draft genome sequence for strain 930029 has been deposited at GenBank under the accession no. CUWS00000000.
